# A Closed-Loop Digital Health Tool to Improve Depression Care in Multiple Sclerosis: Iterative Design and Cross-Sectional Pilot Randomized Controlled Trial and its Impact on Depression Care

**DOI:** 10.2196/52809

**Published:** 2024-03-15

**Authors:** Kyra Henderson, Jennifer Reihm, Kanishka Koshal, Jaeleene Wijangco, Narender Sara, Nicolette Miller, Marianne Doyle, Alicia Mallory, Judith Sheridan, Chu-Yueh Guo, Lauren Oommen, Katherine P Rankin, Stephan Sanders, Anthony Feinstein, Christina Mangurian, Riley Bove

**Affiliations:** 1 Department of Neurology Weill Institute for Neurosciences University of California, San Francisco San Francisco, CA United States; 2 Department of Psychiatry Sunnybrook Health Sciences Centre University of Toronto Toronto, ON Canada; 3 Department of Psychiatry Weill Institute for Neurosciences University of California, San Francisco San Francisco, CA United States

**Keywords:** depression, quality of life, bring your own device, mHealth, closed-loop, clinical trial, multiple sclerosis

## Abstract

**Background:**

People living with multiple sclerosis (MS) face a higher likelihood of being diagnosed with a depressive disorder than the general population. Although many low-cost screening tools and evidence-based interventions exist, depression in people living with MS is underreported, underascertained by clinicians, and undertreated.

**Objective:**

This study aims to design a closed-loop tool to improve depression care for these patients. It would support regular depression screening, tie into the point of care, and support shared decision-making and comprehensive follow-up. After an initial development phase, this study involved a proof-of-concept pilot randomized controlled trial (RCT) validation phase and a detailed human-centered design (HCD) phase.

**Methods:**

During the initial development phase, the technological infrastructure of a clinician-facing point-of-care clinical dashboard for MS management (BRIDGE) was leveraged to incorporate features that would support depression screening and comprehensive care (Care Technology to Ascertain, Treat, and Engage the Community to Heal Depression in people living with MS [MS CATCH]). This linked a patient survey, in-basket messages, and a clinician dashboard. During the pilot RCT phase, a convenience sample of 50 adults with MS was recruited from a single MS center with 9-item Patient Health Questionnaire scores of 5-19 (mild to moderately severe depression). During the routine MS visit, their clinicians were either asked or not to use MS CATCH to review their scores and care outcomes were collected. During the HCD phase, the MS CATCH components were iteratively modified based on feedback from stakeholders: people living with MS, MS clinicians, and interprofessional experts.

**Results:**

MS CATCH links 3 features designed to support mood reporting and ascertainment, comprehensive evidence-based management, and clinician and patient self-management behaviors likely to lead to sustained depression relief. In the pilot RCT (n=50 visits), visits in which the clinician was randomized to use MS CATCH had more notes documenting a discussion of depressive symptoms than those in which MS CATCH was not used (75% vs 34.6%; *χ*^2^_1_=8.2; *P*=.004). During the HCD phase, 45 people living with MS, clinicians, and other experts participated in the design and refinement. The final testing round included 20 people living with MS and 10 clinicians including 5 not affiliated with our health system. Most scoring targets for likeability and usability, including perceived ease of use and perceived effectiveness, were met. Net Promoter Scale was 50 for patients and 40 for clinicians.

**Conclusions:**

Created with extensive stakeholder feedback, MS CATCH is a closed-loop system aimed to increase communication about depression between people living with MS and their clinicians, and ultimately improve depression care. The pilot findings showed evidence of enhanced communication. Stakeholders also advised on trial design features of a full year long Department of Defense–funded feasibility and efficacy trial, which is now underway.

**Trial Registration:**

ClinicalTrials.gov NCT05865405; http://tinyurl.com/4zkvru9x

## Introduction

### Background

Approximately 50% of people with multiple sclerosis (MS) have a depressive disorder, and people living with MS are 2-3 times more likely to be diagnosed with a depressive disorder than the general population [1]. However, depression in MS remains underreported, underevaluated [2], and undertreated [3,4], despite the prevalence of depression in people living with MS, low-cost tools available to screen for depression, and evidence-proven, society-recommended pharmacologic and nonpharmacologic treatment modalities [5,6]. In clinical practice, common barriers to these clinical goals include time constraints during clinical visits (Nelson, M, unpublished data, August 2020), stigma and discomfort discussing psychiatric symptoms [7], insufficient antidepressant medication dose, duration or mechanism of action [8], inadequate attention to other MS symptoms (fatigue, cognitive impairment, and urinary retention) that could interfere with mood and worsen symptom burden [2,9], and patient difficulty following treatments owing to difficulties with access, insurance, or finding specialists close to home [10], as well as the many competing demands on their time. Solutions to address these gaps in reporting, screening, and treatment are needed.

A closed-loop intervention could close these gaps in care and represent a pragmatic approach to address the suboptimal treatment of depression in real-world MS settings. Closed-loop interventions in health care refer to systems that minimize gaps in communication between patients and clinicians; these have been implemented effectively for a number of medical conditions [11-13]. For the purpose of improving depression care in MS, such an intervention would support relevant clinical information flow from patients to clinicians at the point of care and back to patients to support patient-centered care. Such a system would not focus on a specific “one size fits all” treatment or intervention modality (eg, social intervention) or on a specific proprietary mood app, but could support developing a complex plan individualized for each patient’s symptoms, goals, and capability. To accomplish this, the tool should efficiently deliver patient-reported mood symptoms to clinicians in line with the “5 rights” [14] (right information, to the right person, in the right format, through the right channel, and at the right time in the workflow). Further, the tool must promote the behaviors (eg, reporting, screening, treatment recommendations, and following through with timely refills or referral scheduling) that are likely to lead to mood improvements. Finally, this tool should seek to streamline workflows and streamline care within an existing care team (neurologist, nurse, and interprofessional staff) and system of care (informatics, care delivery, and payment structure). Once developed, even after extensive multidisciplinary stakeholder input, a digital health solution must be thoroughly socialized within a health system to increase adoption.

Some prior studies support the feasibility of monitoring patient mood longitudinally in people living with MS as well as the possible effectiveness of a closed-loop approach. There is good concordance between patient reports and clinical depression, supporting the use of patient-reported tools for depression monitoring [15]. The 3-month pilot CoachMS randomized controlled trial (RCT; NCT03335618; n=21; people living with MS; [16]) showed that it was feasible, and acceptable, to monitor patients’ bothersome symptoms (mood, ambulation, and bladder) and to act on them clinically in near real time (coaching patients to address these) [16]. This intervention showed some preliminary efficacy in supporting behavioral change [17,18] likely to address depression, including in one case, recognition of and urgent hospitalization for suicidality. A proposed closed-loop intervention could further deliver data on patient function directly to the clinician at the point of care to support effective response.

### Objectives

This study details a 3-phase endeavor designed to develop a comprehensive, closed-loop system for monitoring and thoroughly treating depression to be tested in real-world clinical settings: MS CATCH (Care Technology to Ascertain, Treat, and Engage the Community to Heal Depression in people living with MS). In phase I, a prototype was developed. In phase II, it was piloted in a clinical setting to obtain preliminary cross-sectional efficacy data on a fundamental premise, namely that visualization of patient mood during the clinical encounter would improve clinical attention to mood during that visit. In phase III, having ensured that the tool was satisfactory to patients and clinicians and that its use did indeed improve attention to depression in the pilot study, the tool was then refined using an extensive process of human-centered design (HCD) [19,20].

## Methods

### Phase 1: Initial Technical Build

#### Overview

Members of the primary research team led earlier engagement efforts that informed MS CATCH development, namely, creating the BRIDGE dashboard concept [21], testing the acceptability of patient-reported outcomes (PROs) in a point-of-care MS clinical dashboard (MS NeuroShare; 19]), and developing a closed-loop reporting system that collects and synthesizes data at the point of care (MS Falls Insight Track; MS-FIT); [22]). Each of these design processes informed the current technical build.

#### BRIDGE

**Figure 1:**
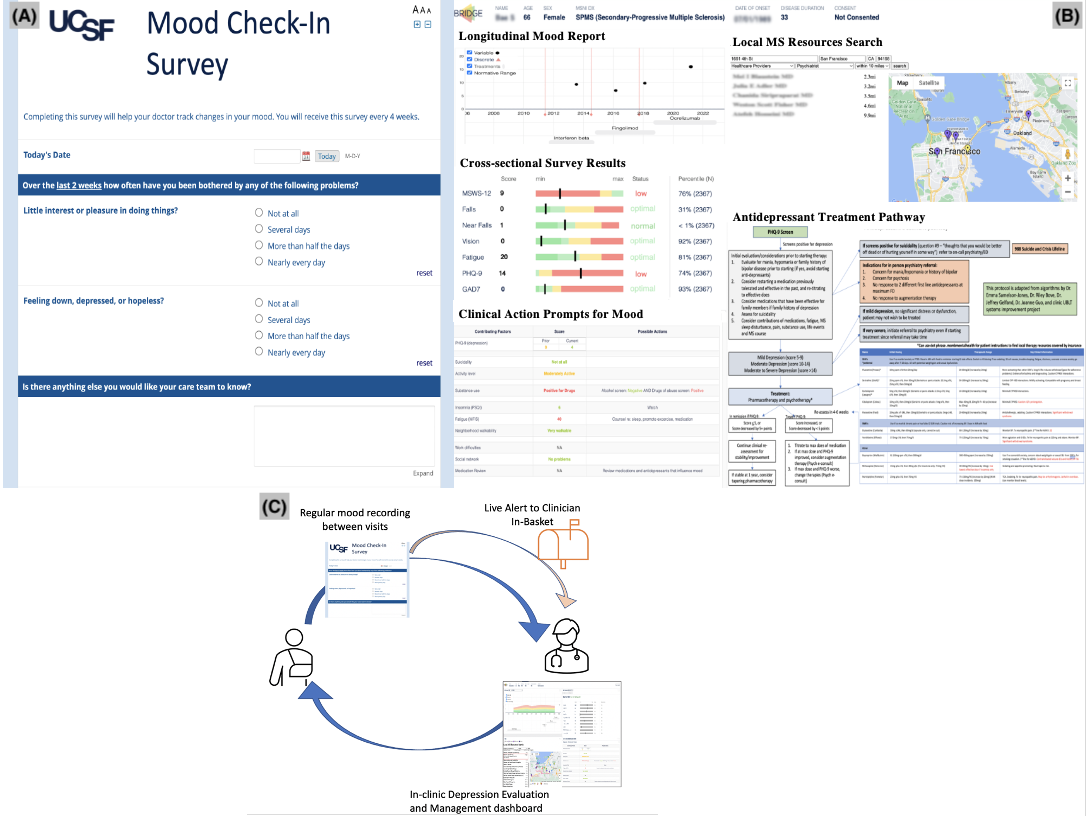
Components of the MS CATCH (Care Technology to Ascertain, Treat, and Engage the Community to Heal Depression in people living with multiple sclerosis) tool, including the (A) patient-facing survey and log, (B) the clinician-facing dashboard, and (C) the closed-loop system, with in-basket alerts for the clinician.

BRIDGE [21] is a technologically scalable, institutionally approved, workflow friendly, cross-disease, modular precision medicine platform [23]. BRIDGE launches from within a patient’s encounter from the Epic electronic health record (EHR) at the University of California, San Francisco (UCSF) using industry standard integration (Figure 1; Table S1 in Multimedia Appendix 1 [24]), delivering a seamless experience for clinicians [21]. BRIDGE was designed using an extensive process of HCD [19,20]. The tool accesses and visualizes data that are currently available in various disparate sources (EHR, patient diaries, and publicly accessible websites), with the substantial innovation that they are presented here in one comprehensive and streamlined format. A disease-specific version of BRIDGE is live within multiple clinics at the UCSF, with the MS clinic BRIDGE used as a point-of-care dashboard for the current project.

#### MS NeuroShare

This closed-loop prototype designed for a collaborative health system integrated a patient-facing PRO app with an EHR-based clinician-facing dashboard [25]. Patient participants cited the perceived value of thinking about and recording information before their appointments, noting how it impacted discussions with their clinicians, adding that coviewing information with the physician put physicians and patients on the same page and promoted a conversation of equals. Clinicians perceived patients’ prospectively collected mood scores as more sensitive, accurate, and comprehensive than a patient’s recall during the visit, and felt that these could improve value and promote clinician engagement [25].

#### MS-FIT Tool

A closed-loop system to prospectively record, report, and prevent falls in people living with MS was designed using HCD in a process analogous to that intended for MS CATCH [22]. MS-FIT includes low-burden regular falls ascertainment that patients can access with one click from any computer, tablet, or device, triggering a clinician’s inbox message for new or serious falls. Clinicians can then access and launch, from the medical record, a comprehensive version of BRIDGE refined to support multimodal falls assessment and prevention [22]. The MS-FIT development process informed the feasibility of a closed-loop approach as well as specific needs and concerns of intended users, people living with MS, and MS care teams.

#### MS CATCH Prototype

The prototype for MS CATCH used in this study was refined for the specific purposes of depression monitoring and treatment by the study team using the BRIDGE and MS-FIT technical scaffolding, and informed by the MS NeuroShare and MS-FIT design processes and experiences. The MS CATCH prototype used in this study is shown in Figure S1 in Multimedia Appendix 1.

### Phase 2: Proof-of-Concept Pilot Randomized Controlled Trial

#### Research Setting and Participants

##### Design

This was a cross-sectional, randomized controlled pilot study.

##### Setting

The primary clinical setting was the UCSF Center for MS and Neuroinflammation, which specializes in providing care to >5000 adults with MS annually, and has an extensive track record of pivotal trials for MS [26,27], remote monitoring [28,29], and treatment [16].

##### Participants

MS clinicians were invited to participate in the study during the center’s monthly research meeting, and a convenience cohort of 6 clinicians was enrolled using a signed electronic consent form. Then, from the participating neurologists’ practices, adult patients with an MS diagnosis scheduled for upcoming in-person and video neurology visits were contacted before the clinic visit by the study coordinator via email and phone call. Interested patients were scheduled for an enrollment visit whether in-person, or via telephone, or Zoom (Zoom Video Communications, Inc; an institutionally approved, Health Insurance Portability and Accountability Act–secure, televideo platform). During this visit, they provided detailed informed consent via DocuSign (DocuSign, Inc). Once patients signed the informed consent form, they were asked to complete a series of PROs, including the 9-item Patient Health Questionnaire (PHQ-9) scale, via a secure REDCap (Research Electronic Data Capture; Vanderbilt University) link. Patients with scores of ≥5 (mild depression: 5-9, moderate: 10-14, moderately severe: 15-19, and severe: 20-27) met the study criteria and were then enrolled in the pilot study. All participants were computer literate. Enrollment was capped at 50 individuals meeting criteria. Full inclusion criteria are summarized in Table S1 in Multimedia Appendix 1.

#### Study Procedures

The PRO data obtained from the patient participants populate the BRIDGE dashboard in real time. Patient visits were randomized 1:1 using a simple randomization scheme in which the clinician was asked by the study team to launch BRIDGE during the participant’s visit. For those who did not use BRIDGE, the visit proceeded as a usual clinical visit. Clinicians were reminded to launch BRIDGE via text or email before the patient appointment when applicable, and neither clinician nor patient participant were blinded to the intervention. Patients continued to be recruited until a target (n=50) had met the inclusion criteria and were seen in the clinic. For participants with PHQ-9 scores of ≥19, even if their clinician was not asked to use BRIDGE, the clinician was informed of their score near the conclusion of the clinic visit to ensure safe treatment of the patient. At the conclusion of the study visit, both clinician and patient feedback regarding the tool were solicited. During the proof of concept pilot, MS CATCH was used once during the visit in real time. For the full RCT, the patient-facing mood survey will be completed monthly, as described in the results for phase III.

#### Trial Outcomes

##### Usability: Participant Feedback on Tool

At the end of the study visit, patients were asked to provide feedback on the tool. There were 2 main prompted questions: “Tell us (with illustrative examples) of what worked well and what didn’t, with regards to BRIDGE, during your appointment.” This question was asked to both patients and clinicians, whereas “Please explain in detail, why it was or wasn’t useful for you to report your mood symptoms before the visit” was just addressed to the patients. Then, 2 questions informed from the System Usability Scale [30,31] and the health IT usability evaluation model [32] were completed, assessing likeability and perceived usefulness of the tool.

##### Efficacy: Effect of the Tool on Attention to Depression

At the end of the study, each clinical encounter was reviewed by a team member with an eye to whether mood was mentioned during the clinical visit. After the review was completed by one team member, another team member blinded to the treatment assignment audited the report. Only the second team member was blinded to treatment assignments. Any disagreements were resolved by a third party.

#### Ethical Considerations

This study was approved by the UCSF institutional review board (#18-26148).

#### Statistical Analysis

The participant demographics and usability outcomes were analyzed using descriptive statistics. The main efficacy outcome, percentage visits where depression was mentioned in the clinic notes, was compared for visits with and without the use of MS CATCH using chi-square analysis [33].

### Phase 3: MS CATCH Tool Refinement (Stakeholder Engagement and Intended User Input Over 6 Months)

#### Study Design

**Figure 2:**
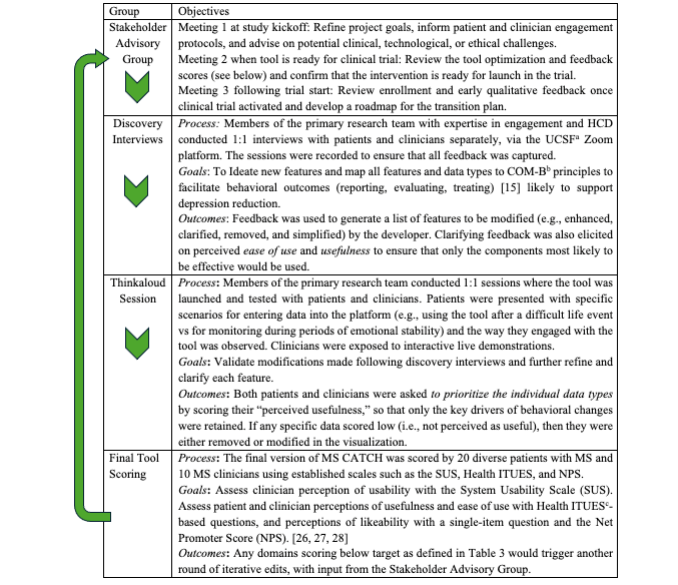
The sequence of iterative feedback and human-centered design (HCD) development leading to tool optimization over 9 months, including activities, participants, and outcomes. COM-B: capability, opportunity, and motivation model for behavioral change; Health ITUES: Health IT Usability Evaluation Scale; MS: multiple sclerosis; MS CATCH: Care Technology to Ascertain, Treat, and Engage the Community to Heal Depression in people living with multiple sclerosis UCSF: University of California, San Francisco.

HCD is a process that holds at its center the needs of the intended users. During the MS CATCH refinement phase, the overall goal was to fine-tune and optimize the intervention by partnering with patient and clinician users and multidisciplinary stakeholders in a sequence of iterative feedback sessions to inform and validate design decisions (Figure 2). To our knowledge, features assembled into the MS CATCH prototype had never before been available at the point of care in a clinically actionable format for depression treatment in MS. It was important to verify that the workflow and displays promoted behavioral change likely to improve depression care and to simplify the display into the features most likely to drive adoption of the tool and support depression care. Here, the “capability, opportunity, and motivation model for behavioral change” (COM-B) [18] was used. COM-B is a hub in the center of Michie’s behavioral change wheel [18] and can help inform a patient’s intention to engage in a planned behavior, which is considered the best predictor of that behavior [34,35]. The COM-B approach, and individual intervention functions, have been successfully applied to facilitate behavioral change in people living with MS [17].

To maximize the likelihood that the tool would be adopted and effective, it was evaluated using the health IT usability evaluation model [32]. This model integrates multiple usability theories including the Technology Acceptance Model and evaluates both subjective and objective outcomes. Although refining the critical data and visualization elements, as well as technological and clinical workflow aspects, the 4 key variables proposed by Mathews et al [36] for digital health tool validation were examined to determine whether the tool (1) reflects HCD principles and (2) is likely to engage patients. These factors include usability, effectiveness, learnability or ease of use, and likeability. Usability was defined using the System Usability Scale, learnability and ease of use were measured with a subset of the Health Information Technology Usability Evaluation Scale–based questions, and likeability was assessed with a single Likert scale question: “Do you like the tool?” and the net promoter score (NPS). Effectiveness was determined based on the pilot study visit notes outcomes.

#### Sequential Methods With Iterative Technological Modifications

Over a 9-month period between September and June, sequential activities were conducted as outlined in Figure 2.

#### Stakeholder Advisory Group

This group was convened and led by members of the primary research team. The primary research team comprised investigators with a track record of collaboration as well as expertise in both scientific and technological aspects of this project, including MS clinical trials (RB), digital tools to evaluate and treat cognition and mood in MS [16] (RB and AF), psychiatric care for underserved populations and innovations in treatment of psychiatric conditions in diverse settings (CM) [37-44], implementation science (CM), statistics (Ann Lazar), patient engagement (RB and JR) [20,21,25], HCD of digital tools (JR, RB, NS, and NM) [20,21,25], and launching an institutionally and technologically sophisticated, scalable, cross-disease platform from the EHR at UCSF (RB and BRIDGE team). The stakeholder advisory group included the research team listed above, as well as a patient champion JS, patient advocacy group leader Linda Glassel, National Multiple Sclerosis Society, Northern California Chapter President, MS nurse expert AM, Registered Nurse, social worker MD licensed independent clinical social worker, and MS neurologist CYG.

#### Participants

MS clinicians and other experts (degrees including Doctor of Medicine, Nurse Practitioner, Registered Nurse, and Masters in Social Work) were identified within the UCSF MS clinic and the investigators’ broader professional network. Adults with MS were recruited from the UCSF MS center by the study team (convenience sampling). All participants provided informed consent to test the tool and provide preliminary data. Furthermore, 31% (10/32) of the patients participated in the phase II pilot study.

#### Ethical Considerations

Activities during this phase (HCD phase) were approved by the UCSF institutional review board (#22-36620).

## Results

### Phase 1: Initial Technical Build

The MS CATCH prototype used for the current phase II and phase III study phases, informed by prior HCD processes (BRIDGE, MS NeuroShare, and MS-FIT), is shown in Figure S1 in Multimedia Appendix 1. A patient-facing mood survey was selected to be administered via a secure REDCap link that could be accessed on any device with Wi-Fi or cellular data capabilities. The validated, self-administered PHQ-9 scale was selected because it assists clinicians in objectively assessing the severity of depression. It has sensitivity and specificity values of 88% and contains 9 criteria that comprise a diagnosis of depressive disorder per the *DSM-IV* (*Diagnostic and Statistical Manual of Mental Disorders* [Fourth Edition]) [45]. This mood survey was then connected to the BRIDGE platform so that it could be visualized at the point of care and interpreted in BRIDGE in addition to the other elements of the patient’s MS history, as well as a list of resources including psychotherapy and psychiatry, available in the patient’s home area (California only). The PHQ-9 survey also triggered a clinician’s inbox message for worsening mood. The mechanism by which MS CATCH is postulated to work is by facilitating communication flows that are likely to support intended users’ COM-B behaviors (patient: reporting and follow through and clinician: evaluating and recommending) likely to lead to symptom improvement. With regard to usability, the intended users, that is, both clinicians and patients, can each access it with one click.

### Phase 2: Proof-of-Concept Pilot Randomized Controlled Trial

#### Participants

Of the 200 patients contacted, 106 (53%) agreed to participate in this study. Of these, 50 (47.2%) met the inclusion criteria of a PHQ-9 score >4 and were cared for by 6 participating physicians. Distribution of PHQ-9 scores was as follows: 52% (26/50) mild depression (5-9); 32% (16/50) moderate depression (10-14); 10% (5/50) moderately severe depression (15-19); and 6% (3/50) severe depression (20-27). Patients were then randomized to visits where the BRIDGE dashboard was used (24/50, 48%) or not used (26/50, 52%). All participants were contacted before their clinical visit, which was conducted between May and December, 2021. This visit was where the MS CATCH intervention was used. The cohort included 40 (80%) females and 10 (20%) males, aged 25-75 (mean 50.46, SD 13.0) years, including individuals with all MS subtypes (32 relapsing remitting MS, 9 primary progressive MS, 1 progressive relapsing MS, 6 secondary progressive MS, 1 unspecified, and 1 MS likely).

#### Tool Usability

The mean *likeability* score was 4.3/5 for patients (93% of the patient participants agreed or strongly agreed that they liked the tool) and 4.4/5 for clinicians (100% of the clinicians agreed or strongly agreed that they liked the tool).

For *perceived usefulness* [30,31], among clinicians, 100% of them reported that the tool increased their attention to the patient’s mood, *particularly for patients with mild to moderate symptoms* that might otherwise have been missed. Clinicians provided some specific feedback on how to further customize the dashboard, suggesting that it would be better to have the categorical components of the PHQ-9 rather than just the raw score (3/6, 50%).

Among patients, 71% (20/28) reported feeling that it was useful to report their mood-related symptoms before the visit. In qualitative feedback, many patients reported that filling out the mood survey ahead of time gave them the opportunity to reflect on their own feelings: “It set the tone, upfront, about how I was feeling,” “It’s helpful for me to think about assessing my mood in a structured way,” “was useful because it made me stop and reflect,” and “it opened the door to explain how...new MS symptoms are affecting my mood.”

#### Tool Effectiveness

For the patients with a PHQ-9 score of ≥5, visit notes documented a discussion of depressive symptoms in 75% of the visits where MS CATCH was used versus 35% of the visits where it was not used (N=50; *χ*^2^_1_=8.2; *P*=.004; Table 1). One patient was referred to a psychiatrist for suicidal ideation.

**Table 1:** MS CATCH (Care Technology to Ascertain, Treat, and Engage the Community to Heal Depression in people living with multiple sclerosis) pilot randomized controlled trial cross-sectional effectiveness. Visit notes documented a discussion of depressive symptoms in 75% of the visits where MS CATCH was used versus 34.6% of the visits where it was not used (N=50; χ21=8.2; *P*=.004).

	MS CATCH tool used (n=24), n (%)	MS CATCH tool not used (n=26), n (%)	Total (n=50), n (%)
Mood discussed	18 (75)	9 (35)	27 (100)
Mood not discussed	6 (26)	17 (74)	23 (100)

### Phase 3: MS CATCH Tool Refinement

#### Study Participants

Overall, 45 individuals provided feedback on the tool during successive phases of development. The results are summarized in Table 2.

**Table 2:** Demographic information for each round of interviews during the MS CATCH (Care Technology to Ascertain, Treat, and Engage the Community to Heal Depression in people living with multiple sclerosis) intervention refinement.

Phase (cohort, round #)	Sample size	Age range^a^ (years)	Sex (females), n (%)	Clinical context
Discovery interviews: Patients	5	30-57.6	3 (60)	MS^b^
Discovery interviews: Clinicians	5	N/A^c^	4 (80)	MS (MD^d^, NP^e^, and MSW^f^)
Think-alouds: patients	7	30.4-66	6 (86)	MS
Think-alouds combined with final tool testing: Clinicians	Think-alouds: 5; tool testing: 10	N/A	6 (60)	MS
Final tool testing: Patients	20	29-63	15 (88)	MS

^a^If applicable.

^b^MS: multiple sclerosis.

^c^N/A: not applicable.

^d^MD: Doctor of Medicine.

^e^NP: Nurse Practitioner.

^f^MSW: Masters in Social Work.

#### Discovery and Think-Aloud Sessions: Selected Findings

Detailed findings and scores from each round of engagement, as well as iterative development steps, are presented in Multimedia Appendix 1 and summarized here. During the *discovery interviews (N=5 patients, N=5 clinicians),* each tool component was found to be useful and usable. A number of features and data types were identified that mapped to the COM-B principles of behavior change, and these resulted in design changes and interventions promoted to address potential boosters or blockers (Table S2 in Multimedia Appendix 1). For example, patients universally felt that the PHQ-9 should be delivered monthly if not more often, despite concerns by the study team that it would be too burdensome. The patient-facing survey was edited to support free-text entry and addition of language at end of survey informing patients who scored >15 to seek care urgently, as their care team may not see the score for 36 hours, and instructing patients to seek immediate care if experiencing a mental health crisis. During the *think-aloud sessions (N=7 patients, N=5 clinicians),* some patients articulated concern for how answers might be interpreted by their clinicians, for example, concern about sending up unnecessary “red flags” with their survey answers as symptoms addressed on the survey can overlap with MS symptoms. Relatedly, the free text box proved to be a popular design feature as it presents an opportunity to offer context for potentially concerning or MS symptom-related survey answers. Additional features were noted to be of value; for example, *neighborhood walkability* was specifically identified by patients as useful context for their clinicians to have within the clinical decision support view. Patients also universally expressed interest in having dashboard views available in a summary display for reference following appointments. All surveyed participants preferred an electronic summary format distributed through the patient portal (MyChart) as opposed to an alternative format that was not integrated with their EHR portal. Clinician feedback led to a reduction in the visual “wordiness” of the original clinical decision support widget. One decision that could not be accommodated was the ability to include patient insurance information, which substantially influences prescribing choices and referral decisions, as there is unfortunately no “ground truth” source for this information.

#### Final Changes Made to Tool Before Scoring

The patient survey was further developed to include an in-survey pop-up directing patients who screened positive for suicidal intent to seek immediate medical care (with contact information), a catalog of mental health resources at the end, and a companion patient-facing mood tracker displaying the patient’s longitudinal mood survey results. On the dashboard side, the clinical decision support screen was further refined to be less “visually overwhelming.”

#### Qualitative Feedback on the Closed-Loop System

Clinicians’ qualitative feedback on the potential impact of MS CATCH on the care delivery experience and quality of conversations with patients and caregivers ranged from appreciation for the intervention’s holistic approach to concern that limited visit times will constrain the dashboards’ seemingly limitless potential.

- “Patients will feel empowered to make decisions using the information.”
- “The value comes from physicians being able to see the patient’s trajectory, and from patients being able to have a longitudinal view.”
- “It will allow me to come into visits with more of a plan.”
- “While [BRIDGE] may not be useful for every problem, it can be valuable for high stakes symptoms (depression, falls).”
- “Feel like it gives me more to offer my patients.”
- “Reinforces what we should do.”
- “Helps to have an objective measure of symptom worsening. Is more reliable than past notes.”

#### Description of the Final MS CATCH Prototype

The final prototype is displayed in Figure 1*.* The patient-facing survey is easily accessible via email. Along with receiving the short monthly survey via email, the patient’s response history and resources relevant to depression self-management can be viewed at the end of the survey (Figure S2 in Multimedia Appendix 1). PHQ-9 scores of 15 or higher or any suicidal intent trigger an alert to the patient’s MS clinician’s EHR in-basket. This is a critical feature allowing for timely follow-up within the usual care workflow, including an urgent referral to psychiatry. The clinician-facing dashboard (Figure 1) has a comprehensive mood evaluation and treatment dashboard that launches from the EHR. This dashboard can be copied and pasted into the clinical notes as well as the patient’s after visit summary to be accessed at the end of the visit and between visits. The tool uses a closed loop system, diagramed in Figure 1, which integrates the patient survey, in-basket messaging, and clinical dashboard tools.

#### Final Tool Scoring

A convenience sample of adults with MS (n=20) and clinicians (n=10) participated in the final tool testing. Of the participating clinicians, 5 (50%) practiced at external (non–UCSF-affiliated) institutions, and 5 (50%) were affiliated with UCSF. The target and achieved results are presented in Table 3.

Patient assessment ratings exceeded the goals in approximately all categories. Despite a patient NPS of 50, which is considered one point below excellent, slightly fewer (75%) than the goal of 80% agreed or strongly agreed that the tool was likeable (mean 4.25, SD 1.12). Of those (n=5) who scored the tool ≤4 with regard to likeability, approximately all (n=4, 80%) were neutral (score=3). Reasons varied from lack of personal applicability, “Mood symptoms are due to MS, not depression,” and “I have a lot of anxiety, but not depression”; to survey design, “I don’t like the multiple-choice options”; and to formatting, “Font is small.” Concrete follow-on actions taken by the development team in response to this feedback were as follows:

- Increasing the default survey font size, in addition to giving patients the option to adjust the font independently.
- Adding a note above the free text box indicating it can be used to add comments or clarify answers.
- Coaching physicians on how to communicate with patients about the survey, including how it can help detect anxiety and mood symptoms that may or may not be MS-related.

Similarly, clinician ratings exceeded the goals in approximately all categories. A clinician NPS of 40 indicated strong favorability in terms of recommending to peers. Perceived usefulness was slightly under goal at 95%. Although all but 1 clinician agreed or strongly agreed that the tool was useful (mean 4.35, SD 0.75) and 1 rated the tool ≤4 citing concerns about limited bandwidth and lack of financial incentives or reimbursable mechanisms for clinicians to address mood between clinical encounters. This feedback echoes concerns noted during discovery interviews about visit time limitations and the need for complementary workflows to ensure that patient mood issues can be monitored in a timely fashion, with the assistance of multidisciplinary staff (registered nurse and masters in social work) and elevated to physicians as needed.

**Table 3:** Final tool scoring by patients (N=20) and clinicians (N=10): Likeability and Usability. Prespecified targets and achieved results are presented; bolded results are at or above target.

	Tool	Patients (N=20)			Clinicians (N=10)	
		Target	Result	Target		Result
Likeability	Single question (“Do you like this tool?”) graded on a Likert scale^a^; metric: score >4 (agree or strongly agree)	80%	75%	80%		100%
Likeability	Net promoter score (NPS)^b^: “how likely are you to recommend this tool to another patient with MS/clinician?” [46]	Good: >0; favorable: >20: excellent: >50	50%	Good: >0; favorable: >20: excellent: >50		40%
Usability	SUS^c^: a rapid, valid, scalable industry standard, reliable with small sample sizes	—^d^	—	75 (68 is average)		78 (SD 11.8)
Perceived usefulness	Health ITUES^e^-based questions for perceived usefulness [30,31]; metric: score ≥4 (agree or strongly agree)	80%	88%	100%		95%
Perceived ease of use	Health ITUES-based questions for perceived ease of use [30,31]; metric: score ≥4 (agree or strongly agree)	80%	97%	100%		100%

^a^Likert scale ranging from 1 (strongly disagree) to 5 (strongly agree).

^b^NPS responses (0-10 scale) were calculated by subtracting the percentage of detractors (those who scored 0-6) from the percentage of promoters (those who scored 9 or 10).

^c^SUS: System Usability Scale.

^d^Not available.

^e^Health ITUES: Health IT Usability Evaluation Scale.

## Discussion

Depression is a serious comorbidity in MS that is associated with an increased risk of disability progression [47], yet it remains severely undertreated; by one estimate, two-thirds of individuals meeting the clinical criteria for major depressive disorder receive no antidepressant medications [48]. Depression is often missed altogether by neurologists, even when it is severe and accompanied by suicidal ideation [49,50]. We iteratively and extensively piloted and developed a closed-loop intervention designed to improve reporting, screening, and treatment of depression in a cohort of clinical patients with MS. The preliminary data presented here support our key premise underlying this closed-loop approach [11] that delivering reliable information about a patient’s mood in an interpretable, visual format to the clinician would increase the likelihood that depression was detected (including, in one case, suicidality) and addressed in the clinical encounter. Further, our robust process of HCD and tool evaluation allowed us to maximize the likelihood that this tool would be adopted by ensuring that it met the stringent goals of being likeable, easy to use, and perceived as being effective.

We are not aware of other closed-loop tools developed to support depression care in MS that are integrated with the EHR. Distinct from other mood apps on the market, the patient self-report app directly connects to the clinician’s in-basket so that the information can be efficiently streamlined. In full clinical use, the patient-facing survey would be distributed once per month, and clinicians will not be reminded to launch BRIDGE. Studies have generally shown that integration between psychiatric and nonpsychiatric care in the EHR is often low, and better integration is associated with improved psychiatric care [51]. In this study, we showed that viewing patients’ self-reported mood at the point of care improved attention to depression in the clinical notes. This of course does not inform follow through on this intention. To that end, our 3-month pilot CoachMS RCT (NCT03335618; 21 people living with MS) showed that monitoring and responding to patients in real time [16] was feasible, acceptable, and showed preliminary effects on behavioral change [17,18] suggesting that sustained monitoring and feedback can promote behavioral change [16].

After development, implementation into clinical workflows represents the final goal of health-related tools. From prior experience in developing platforms for the clinical encounter, it was clear that socialization of a tool is a critical step. For patients, knowing that information about their mood will tie back to their care team and translate into improved attention to their care is an important component of tool use, as is the ability to self-monitor using the survey results. Furthermore, direct actionable steps were implemented based on patient feedback, such as providing a list of resources at the end of the survey to reduce the activation energy required to access resources during a depressive episode. Similarly, clinicians are more likely to use and recommend a tool if they know that patients will have completed their surveys [25] and are more likely to want access to patient-generated data if it is clinically actionable. Integrating comprehensive management resources will support individualization of the treatment plan rather than “one size fits all” treatment interventions.

Many treatment trials for depression have to date focused on “one size fits all” approaches testing a specific intervention or combination of interventions. Novel approaches tested within MS include sequential blocks of medications [52] as well as combinations of behavioral and pharmacological interventions [53]. Extending beyond the usual screening and care components (such as asking about moods retrospectively), MS CATCH will comprehensively visualize the other functions contributing to depression, including other conditions (eg, sleep) and unmet social needs (eg, substance use counseling, neighborhood unsafe for walking exercise). These will support *customized action prompts* to help patients self-manage, and clinicians will tailor treatments, anticipate challenges, and make social and behavioral health referrals.

Some limitations were evident in the design and compromises had to be made. For example, the PHQ-9, which is widely adopted as a screening tool, may not be particularly specific. For example, in response to the “constant bother” question, one participant responded “MS is a constant bother.” However, this tool was selected because of its brevity, extensive validation across multiple conditions including MS, and because it comprised the 9 criteria that are identified in the diagnosis of depressive disorder per the *DSM-IV* [45]. Another limitation is the lack of information regarding insurance acceptance by mental health professionals. Unfortunately, this limits actionability in terms of referrals to mental health professionals in the United States. For the resources map, we chose to focus on mental health professionals near the patient who were considered to have expertise for individuals with MS, but we also included a link to a website that includes insurance information to allow cross-checking. A third challenge is scalability. Key technological and clinical features were intentionally selected to ensure that the tool’s modular infrastructure could be scaled to other symptoms, conditions, and clinical settings. Technological factors designed to support scalability include (1) the quality and content of static visualizations that can be disseminated broadly regardless of a clinic’s technology and (2) optimizing the industry technological standards used for the build so that the code can be shared by clinicians in other health settings (eg, other MS centers using Epic EHR). However, the integration of the build into other health systems ultimately depends on governance and motivation internal to that system.

MS CATCH is a comprehensive, low-burden, closed-loop platform designed to reduce depression severity and prevalence in people living with MS by supporting real-time communication and alerts, shared decision-making, and action prompts for comprehensive, personalized interventions. The research findings summarized here suggest that MS CATCH scores well on likeability and usefulness scales and that it improves attention to mood in individual clinic visits. In a complementary pilot study, we previously showed that remote monitoring of mood can lead to timely intervention [16]. Altogether, these findings support the results of a planned randomized single-center clinical trial evaluating its effect on behaviors that support depression reporting, ascertainment, and care. A similar model can be used to improve other clinical symptoms in MS and for managing other chronic medical conditions with high prevalence of depression.

## Appendix

Multimedia Appendix 1 Supplementary tables and figures.

Multimedia Appendix 2 CONSORT-eHEALTH checklist (V 1.6.1).

## Abbreviations

COM-B: capability, opportunity, and motivation model for behavioral change
DSM-IV: Diagnostic and Statistical Manual of Mental Disorders (Fourth Edition)
EHR: electronic health record
HCD: human-centered design
MS CATCH: Care Technology to Ascertain, Treat, and Engage the Community to Heal Depression in people living with multiple sclerosis
MS: multiple sclerosis
NPS: net promoter score
PHQ-9: 9-item Patient Health Questionnaire
PRO: patient-reported outcome
RCT: randomized controlled trial
REDCap: Research Electronic Data Capture
UCSF: University of California, San Francisco
