# Supplementary material for: A Closed-Loop Digital Health Tool to Improve Depression Care in Multiple Sclerosis: Iterative Design and Cross-Sectional Pilot Randomized Controlled Trial and its Impact on Depression Care
Source: JMIR Form Res. 2024 Mar 15;8:e52809. doi: 10.2196/52809 (PMC10980989; doi:10.2196/52809)
Supplement: Multimedia Appendix 1 [file formative_v8i1e52809_app1.pdf]

Supplementary Table 1. Major inclusion and exclusion criteria for the pilot study participants

Inclusion criteria:	*Exclusion criteria*:
• Diagnosis of MS (relapsing or progressive) by 2017 McDonald Criteria[56]<br>• Ages 18 -80<br>• PHQ9 score of 5-19<br>• Any MS therapy, or no treatment	• Cognitive dexterity or visual impairment that, in the opinion of the study neurologist (RB), would put the participant at risk or limit their ability to adhere to the study protocol<br>• Inability to provide informed consent<br>• Psychotic disorders: bipolar disorder, schizophrenia, schizoaffective disorder

Supplementary Table 2. The relationship between tool features and COM-B principles of behavioral change.

A. Results of discovery interviews with patients mapping tool features to COM-B principles of behavioral change.

COM-B Domain	Tool feature – desired behavior	Patient feedback	Booster or blocker	Interventions to address identified or potential blockers
Capability - physical	Survey – mood reporting	Easy to adjust survey font size	Booster	N/A
	Survey – mood reporting	100% of patients reported owning a device (smartphone or computer) on which they receive email	Booster	Enablement – ensure patients have access to device before fielding survey
	Summary – depression treatment	100% of patients reported having access to the patient portal to receive after visit summaries	Booster	Enablement – ensure patients have access to patient portal before distributing summary
Capability - psychological	Survey – mood reporting	100% of patients reported the ability to use a device (smartphone or computer) to receive email; average confidence using the device 4.6 (5 = very confident).	Booster	Education – ensure patients have instructions for accessing survey link via email, if needed
	Dashboard and Summary – mood evaluation (discussion with clinician) and treatment	Agree (mean score >4) all views are easy to understand.	Booster	Education – add plain language definitions of all survey names
	Dashboard and Summary – mood evaluation (discussion with clinician) and treatment	Agree (mean score >4) information is useful for following views:<br>• Longitudinal PROs<br>• Cross sectional PROs<br>• Resource map	Booster	Education – add plain language definitions of survey names
	Dashboard and Summary – mood evaluation (discussion with clinician) and treatment	Neutral (mean score <4) clinical decision support view is useful	Blocker	Education – advise clinicians to use discretion before including view in patient summary
Opportunity	Survey – mood reporting	100% of patients reported daily access to WiFi	Booster	Enablement – ensure patients have access to WiFi before fielding survey
	Survey – mood reporting	Frequency of assessments every 4 weeks is acceptable	Booster	N/A

Motivation - reflective	Survey – mood reporting	80% feel it is very important to pay attention to and do things to manage mood	Booster	Education – include survey introduction language that includes explanation of survey purpose
	Survey – mood reporting	60% feel it is very important to share how their mood is with their care team	Booster	Education – include survey introduction language that includes explanation of survey purpose; reinforce by showing dashboard during clinical encounters
	Survey – mood reporting	100% feel their care team can do things to help them manage mood	Booster	Education – include survey introduction language that includes explanation of survey purpose
Motivation - automatic	Survey – mood reporting	20% reported cultural barrier to mood reporting (topic is not discussed)	Blocker	Enablement – ensure summary is available in electronic and print formats so that it can be easily shared with family
	Survey – mood reporting	More than half reported concern that answering questions honestly would send up a false “red flag” about their mood, resulting in alarm for clinician	Blocker	Enablement – add free text box to PHQ2 and PHQ9 so patients can provide context for survey answers

B. Results of discovery interviews with clinicians mapping tool features to COM-B principles of behavioral change.

COM-B Domain	Tool feature – desired behavior	Clinician feedback	Booster or blocker	Interventions to address identified or potential blockers
Capability	Dashboard – mood evaluation	Clinicians all have access to dashboard during clinical encounters	Booster	N/A
	Survey – mood reporting	Clinicians feel PHQ-9 is agnostic way of raising mood topic	Booster	N/A
Opportunity	Dashboard – mood evaluation	Limited visit time is the universal factor limiting clinicians’ opportunity to address mood-related issues; mood discussions can be sidelined if the patient has other pressing issues to cover	Blocker	Education – consider informing patients that a separate appointment can be scheduled to address mood<br><br>Enablement – redesign the clinical decision support screen to be easier to digest

				at a glance; with fewer recommendations
	Survey – mood reporting (in-basket messaging)	Clinicians desire a formal clinic protocol in place to set expectations around responses to urgent mood reporting situations	Blocker	Enablement – include language informing patients who score >15 to seek care urgently; alert that care team may not see score for 36 hours<br><br>Education – consider informing patients that a separate appointment can be scheduled to address mood
	Survey – mood reporting	Clinicians are concerned about getting too many "yes" alerts to #9 given prevalence of passive SI within MS population	Blocker	Enablement – add free text box to PHQ9 so patients can provide context for survey answers
Motivation	Dashboard – mood evaluation	Clinicians all reported feeling confident asking patients about mood, but confidence can vary based on how well they know the patient and availability of resources to act on the information.	Blocker	Enablement – include adequate resources in dashboard<br><br>Training – ensure clinicians routinely open BRIDGE to normalized mood discussions during appointments
	Survey – mood reporting	Clinicians feel it is very important to be informed about patient mood because it can impact other symptoms, is treatable, and impacts QoL.	Booster	Education – encourage patients to report mood by stressing symptoms can be addressed
	Dashboard – mood evaluation and treatment	Clinicians are motivated address mood issues, treat mood, and refer patients to supports out of a desire to care for patients.	Booster	Enablement – include adequate resources in dashboard
	Dashboard and Summary – mood evaluation and treatment	Clinicians report concerns about treatment efficacy knowing resources are scarce from both the patient navigation assistance and mental health provider perspectives.	Blocker	Enablement – include smart phrase instructions to guide patients on referral follow-through and system navigation; ensure adequate resources included in dashboard
	Summary – mood treatment	Clinicians report giving patients the option of making dashboard views available is "not harmful," but they are	Blocker	Education – add plain language definitions of all survey names; advise clinicians to use discretion

		not sure how much patients will use them, with possible exception of resources; perception that more useful for higher literacy patients		before including view in patient summary

Supplementary Results: Findings from the Discovery interviews and Thinkaloud Sessions

Discovery Interviews (N=5 patients, 5 clinicians)

Participants' initial evaluation of ease of use (patients only) and usefulness (patients and clinicians) of the various prototype features are summarized in *Table 3*. All tool components were easy to understand. The clinical decision support dashboard view scored <4 by both patients and clinicians. Clinicians felt it was "too busy" as designed and that the overwhelming number of recommendations did not feel "actionable" given limited appointment times. Patients felt that while the view's color-coded status section was helpful, it was comparatively less meaningful than the cross-sectional display which shows current status within the context of a green/yellow/red continuum. The resource map had a high mean perceived usefulness among patients (4.8, SD 0.45), but a lower mean perceived usefulness (2.4, SD 0.89) and the lowest mean importance rating among clinicians (1.6, SD 0.89). Specifically, clinicians noted the importance of providing information about insurance for any resources in order to make the information actionable (prescribing choices and referral decisions) and patients (ability to follow through). Patients acknowledged these challenges but still appreciated the user-friendly design and having "a place to start" with regard to referrals that would reduce the burden of seeking resources when actively experiencing emotional distress. Both audiences noted the need for resources to be current and drawn from reliable sources. A number of features and data types were identified that mapped to the COM-B principles of behavior change, and these resulted in design changes and interventions promoted to address potential boosters or blockers (*Supplementary Table 2*).

Supplementary Table 3. Patient (n = 5) and clinician (n = 5) participants' assessment of the ease of use and usefulness of the various tool components.

	PATIENTS				CLINICIANS	
	**How useful would this information be to you?**		**How easy is this information to understand?**		**How useful would this information be to you?**	
	**Mean**	**SD**	**Mean**	**SD**	**Mean**	**SD**
**BRIDGE DASHBOARD**						
Longitudinal PROs	4.40	0.55	4.20	1.30	4.50	1.12
Cross-sectional PROs	4.00	1.73	4.60	0.55	4.70	0.67
Clinical decision support	3.60	1.67	4.50	0.71	3.90	0.89
Resource map	4.80	0.45	4.95	0.11	2.40	0.89
Antidepressant treatment pathway	-	-	-	-	4.38	0.48
**Mood Survey**						
Your patients' mood survey results	-	-	-	-	4.60	0.89
**In-Basket Alerts**						
In-basket alerts triggered by your patients' mood survey results	-	-	-	-	4.75	0.50

Score 1 (strongly disagree) – 5 (strongly agree)

Thinkaloud Sessions

Prior to the thinkaloud sessions, the prototype was refined in response to the discovery interviews (*vide infra* for tails). The survey was iterated to incorporate changes designed to address identified or potential blockers that emerged from discovery interviews. Changes to the patient-facing features included the addition of free text box at end of PHQ2 and PHQ9 surveys, refinement of introductory language at top of the survey, and section formatting to clearly delineate individual questions from summary question sections. The clinician-facing dashboard was also iterated to incorporate changes designed to address identified or potential blockers that emerged from discovery interviews. Changes included the addition of smart phrase instructions to guide patients on referral follow-through and system navigation; addition of language at end of survey informing patients who score >15 to seek care urgently; alert that care team may not see score for 36 hours and instructing patients to seek immediate care if experiencing a mental health crisis.

Patient Sessions (n = 7).

Patient survey.

During thinkaloud sessions, patients assessed the survey for usability and understandability in different scenarios. They were subsequently shown designs and asked questions about the BRIDGE dashboard and any BRIDGE dashboard screens that they would like to have access to in a summary display following appointments.

Survey feedback.

Patients were universal in their desire to receive the survey at least every four weeks, if not more often. All patients strongly agreed the survey was easy to open using the link (Supp. Table 4). In Scenario A, patients were asked to pretend they had <u>not</u> been bothered by mood-related problems over the last few weeks in order to only receive the PHQ-2 survey. In Scenario B, patients were asked to pretend they had been bothered “several days,” “more than half,” or “nearly every day,” by mood-related problems over the last few weeks in order to receive the PHQ-9 survey. The slightly longer and more complex PHQ9 was still graded as easy to complete and understand relative to the PHQ-2. Some patients articulated concern for how answers might be interpreted by their clinicians, for example, concern about sending up unnecessary “red flags” with their survey answers as symptoms addressed on the survey can overlap with MS symptoms. Relatedly, the free text box proved to be an extremely popular design feature as it presents PwMS who are experienced in recognizing their own symptoms, an opportunity to offer context for potentially concerning or MS symptom-related survey answers.

Supplementary Table 4. The patient responses to ease of use during Thinkaloud sessions (N=7)

	Mean		SD	
How easy was it to open the survey using the link?	5.00		0.00	
	**Scenario A: PHQ-2**		**Scenario B: PHQ-9**	
	**Mean**	**SD**	**Mean**	**SD**
How easy was it to complete the survey?	5.00	0.00	4.93	0.19
How easy were the questions to understand?	5.00	0.00	4.50	0.76

Score 1 (strongly disagree) – 5 (strongly agree)

Dashboard and Summary Display.

When ranking each dashboard view according to how important it was to them, participants during the 23 thinkaloud sessions ranked views as more important (longitudinal PROs and resource map) that had differed from those during the discovery interviews (cross-sectional PROs and clinical decision support), indicating the importance of allowing patients to choose what dashboard images appear in their after visit summary displays. Neighborhood walkability was specifically identified by patients as useful context for their clinicians to have within the clinical decision support view. Patients universally expressed interest in having dashboard views available in a summary display for reference following appointments. All surveyed preferred an electronic summary format distributed through the patient portal (MyChart).

Clinician Sessions (n = 5)

During 23 thinkaloud sessions, clinicians were shown live, interactive demonstrations using sample data to illustrate how various MS CATCH intervention components (patient survey, in-basket messaging, BRIDGE dashboard) work together to form a closed-loop system. Clinicians were asked to rate each of five dashboard views for usefulness and importance, as well as share qualitative feedback about overall completeness, understandability, and potential impact on care delivery.

Dashboard.

Interestingly, the clinical decision support screen was deemed among the most important views (mean 4.2, SD 1.1), along with the longitudinal PROs (mean 5.0, SD 0) and the resource map (mean 4.5, SD 0.87) (Table 5). However, as currently designed, clinicians perceived the clinical decision support screen as less useful (mean: 3.20, SD 1.15) because it was perceived as potentially overwhelming ("wordy") and the guidance too generic. Some felt it would be more useful for newer clinicians. Others worried about accomplishing all recommended tasks during a single visit. In contrast, the longitudinal and cross-sectional PROs, as well as the resource map were perceived as most useful. Critiques of the antidepressant treatment pathway were conflicting. Some reported it included too much information, others said not enough information. Several spoke of a bias to refer to psychiatry rather than prescribe. Echoing discovery interview feedback, nearly all clinicians expressed a desire to incorporate patient insurance information as it influences prescribing choices and referral decisions.

Supplementary Table 5. Clinician responses to usefulness and importance during Thinkaloud sessions (n = 5)

	How useful would each display be to you?		How important is each display to you?	
	**Mean**	**SD**	**Mean**	**SD**
Longitudinal PROs	4.30	0.45	5.00	0.00
Cross-sectional PROs	4.30	1.30	3.60	1.67
Clinical decision support	3.20	1.15	4.20	1.10
Resource map	4.70	0.45	4.50	0.87
Antidepressant treatment pathway	3.60	1.29	2.40	0.55

Score 1 (strongly disagree) – 5 (strongly agree)

Final Changes Made to Tool before Scoring.

Design changes.

In response to feedback received from patients and clinicians during thinkaloud sessions, the tool was further refined. Patient-facing aspects of the tool were iterated to include a catalogue of mental health resources at the end, an in-survey pop-up directing patients who screened positive for suicidal intent to seek immediate medical care (with contact information), and a companion patient-facing mood tracker displaying the patient's longitudinal mood survey results. In response to feedback from clinicians that the clinical decisions support screen was too visually overwhelming, stylistic changes were made to make it less so. Instead of having bright red, yellow, and green filled boxes behind the text, the text colors were changed to a muted red, yellow, and green.

Supplementary Figure 1. The prototype for MS CATCH.

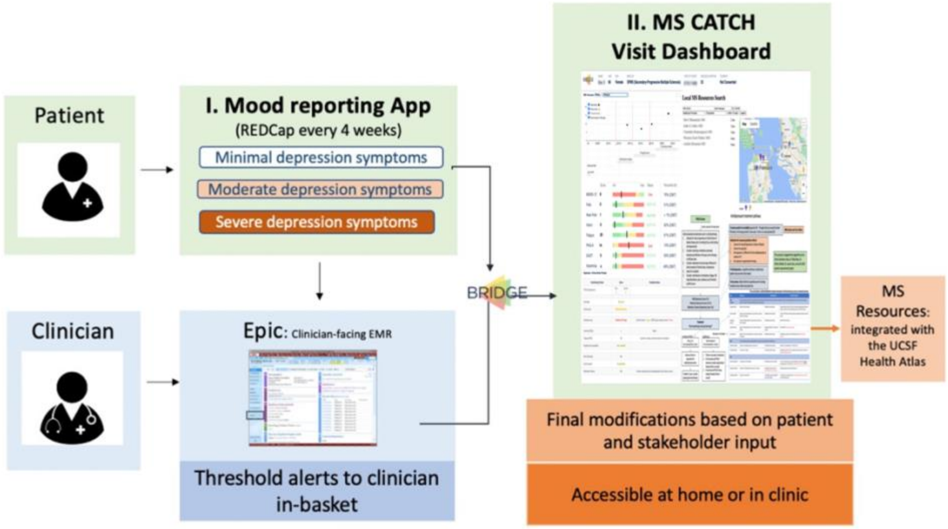

Supplementary Figure 2. The patient's response history and resources relevant to depression self-management can be viewed at the end of the survey.

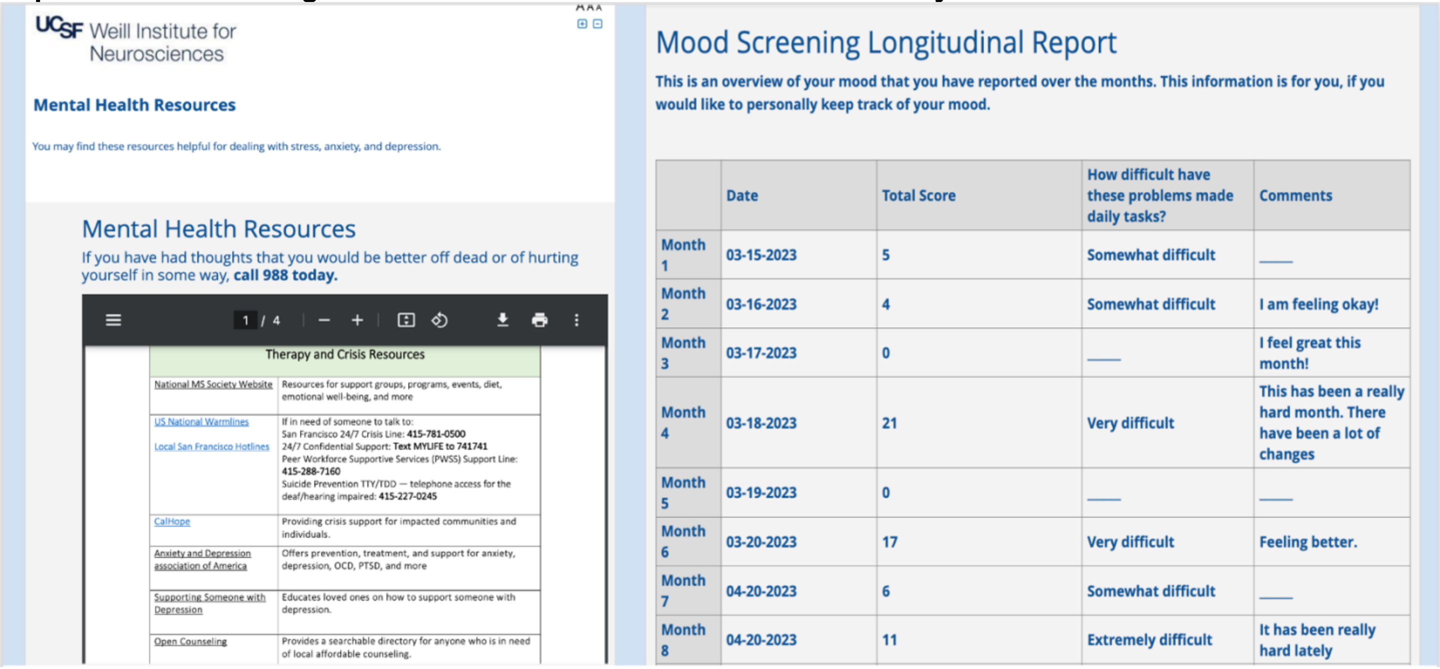
UCSF Weill Institute for Neurosciences

Mental Health Resources

You may find these resources helpful for dealing with stress, anxiety, and depression.

Mental Health Resources

If you have had thoughts that you would be better off dead or of hurting yourself in some way, **call 988 today.**

1 / 4

Therapy and Crisis Resources	
National MS Society Website	Resources for support groups, programs, events, diet, emotional well-being, and more
US National Warmlines<br>Local San Francisco Hotlines	If in need of someone to talk to:<br>San Francisco 24/7 Crisis Line: **415-781-0500**<br>24/7 Confidential Support: **Text MYLIFE to 741741**<br>Peer Workforce Supportive Services (PWSS) Support Line: **415-288-7160**<br>Suicide Prevention TTY/TDD — telephone access for the deaf/hearing impaired: **415-227-0245**
CalHope	Providing crisis support for impacted communities and individuals.
Anxiety and Depression association of America	Offers prevention, treatment, and support for anxiety, depression, OCD, PTSD, and more
Supporting Someone with Depression	Educates loved ones on how to support someone with depression.
Open Counseling	Provides a searchable directory for anyone who is in need of local affordable counseling.

Mood Screening Longitudinal Report

This is an overview of your mood that you have reported over the months. This information is for you, if you would like to personally keep track of your mood.

	Date	Total Score	How difficult have these problems made daily tasks?	Comments
Month 1	03-15-2023	5	Somewhat difficult	____
Month 2	03-16-2023	4	Somewhat difficult	I am feeling okay!
Month 3	03-17-2023	0	____	I feel great this month!
Month 4	03-18-2023	21	Very difficult	This has been a really hard month. There have been a lot of changes
Month 5	03-19-2023	0	____	____
Month 6	03-20-2023	17	Very difficult	Feeling better.
Month 7	04-20-2023	6	Somewhat difficult	____
Month 8	04-20-2023	11	Extremely difficult	It has been really hard lately
